# A review of the assessment and prevalence of sedentarism in older adults, its physiology/health impact and non-exercise mobility counter-measures

**DOI:** 10.1007/s10522-016-9640-1

**Published:** 2016-03-14

**Authors:** Jorgen A. Wullems, Sabine M. P. Verschueren, Hans Degens, Christopher I. Morse, Gladys L. Onambélé

**Affiliations:** Department of Exercise and Sport Science, Institute for Performance Research, Manchester Metropolitan University, Crewe Green Road, Crewe, CW1 5DU UK; Musculoskeletal Rehabilitation Research Group, Department of Rehabilitation Sciences, KU Leuven, Louvain, Belgium; School of Healthcare Science, Manchester Metropolitan University, Manchester, UK; Lithuanian Sports University, Kaunas, Lithuania

**Keywords:** Ageing physiology, Musculoskeletal, Older adults, Physical activity, Sedentary behaviour

## Abstract

This literature review focuses on aspects of sedentary behaviour (SB) in elderly. Since it has been identified as a distinct health risk, independent of physical activity, SB is a significant issue. This is particularly true for an ageing population as evidence shows that older adults (aged ≥65 years) are the most sedentary age group (on average 8.5–9.6 h daily sitting time). Accurate SB assessment is important for understanding this habitual behaviour and its impact. However, SB measurement is challenging, regardless of the method used. Although negative associations of SB in elderly have been reported for several health outcomes, evidence is inconclusive, apart from the evidence on the adverse SB effect on the all-cause mortality rate. Generally, strategies have been proposed to counteract SB, of which breaking prolonged sedentary bouts with at least light-intensity physical activity seems to be the most promising. Overall, further research in elderly is required to increase the evidence and to either support or refute the current findings. Moreover, further research will help to develop informed SB guidelines for an optimal strategy to counteract SB and its health effects in older adults.

## Introduction

Contrary to general perceptions, sedentary behaviour (SB) does not necessarily reflect a lack of physical activity (PA) (Sedentary Behaviour Research Network 2012). Instead, SB is defined as any waking behaviour characterized by an energy expenditure ≤1.5 metabolic equivalent of task (MET) while in a seated or reclined posture (Sedentary Behaviour Research Network 2012). Currently, time spent sitting is increasing in modern societies, presumably linked to activities related to work, leisure or commuting. Previous research has shown that higher sitting time is related to poorer health (Gardiner et al. 2011c; Inoue et al. 2012). Recent health improvement strategies have focused on increasing PA (Kikuchi et al. 2014). While PA contributes to healthy ageing and plays a key role in the prevention of non-communicable diseases and disability, including cardiovascular disease, cancer, metabolic syndrome, mental disorders, musculoskeletal diseases and even all-cause mortality (de Rezende et al. 2014a; Gorman et al. 2014; Gennuso et al. 2015), studies that controlled for PA intensity provide evidence that also (prolonged) SB is an independent determinant of health (Gennuso et al. 2013; Gorman et al. 2014; de Rezende et al. 2014b; Gianoudis et al. 2015). This has led to the proposal of a novel stratagem for reducing health risks through not only increasing PA, but also decreasing SB (Hamilton et al. 2008; Owen et al. 2011).

Recently, the study of SB and its relation to health has become more popular (de Rezende et al. 2014a), but at present most underlying mechanisms by which SB has deleterious health effects remain unknown (Gianoudis et al. 2015). Moreover, existing studies have generally focused on different outcome measures and presented divergent conclusions, making the formulation of a cohesive understanding of the interaction between SB and health, as yet, impossible (de Rezende et al. 2014b). Although SB research shows that older adults (aged ≥65 years) are the most sedentary, this age group has only been studied limitedly (Gennuso et al. 2013; Van Cauwenberg et al. 2014b). This makes it difficult to allow policy recommendations giving detailed information on how to reduce SB in older adults (Harvey et al. 2013). With an ageing population, the increased SB is challenging for both health and social care resources, and better understanding of the relationship between SB and health in the elderly requires more and better-targeted research (de Rezende et al. 2014a). To aid in developing targeted research programmes it is important to identify and summarize current findings of SB in older adults.

Hence, the aim of this review was to describe multiple aspects of SB in older adults, from its assessment, prevalence, physiology, health impact, through to any known potential counteracting strategies.

**Fig. 1:**
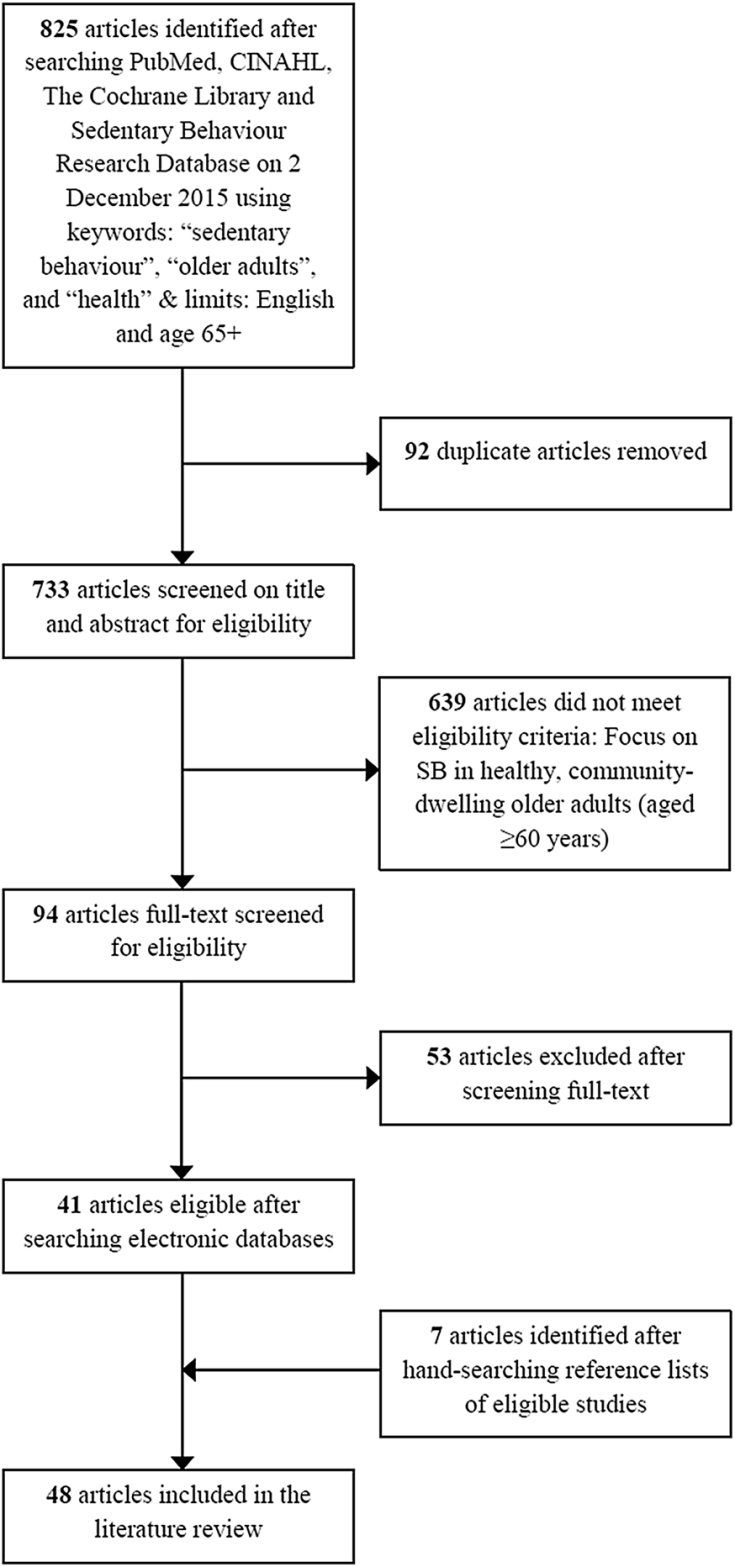
Literature search flow diagram

The strategy used to meet the aims of this literature review was based on a search in four different electronic databases (PubMed, CINAHL, The Cochrane Library and Sedentary Behaviour Research Database) combining the following key words: “sedentary behaviour”, “older adults”, and “health”. Where possible, the following search limits were used: English language and age group 65+. This search (performed on 02 December 2015) identified 825 peer-reviewed articles. All were screened for potential inclusion based first on the title and abstract, and if not excluded, the full-texts were checked for eligibility. Generally, eligible articles focused on SB (or a proxy measure, but not physical inactivity) as a main independent or dependent variable in healthy, community-dwelling older adults (aged ≥60 years) only. In addition to the electronic databases search, reference lists of the eligible articles (n = 41) were hand-searched to identify any missed papers (n = 7) (Fig. 1). Table 1 shows an overview of the 48 included papers, which are fundamental to this review.

**Table 1:** Overview of the 48 included studies after literature search

Data presented in paragraph(s)	Author(s)	Study population	Subjective or objective SB tool	General finding(s)
Original studies				
Assessment of SB	Van Cauwenberg et al. (2014b)	n = 508	Both	Validity for older adults’ self-reported total sitting time against accelerometer-derived sedentary time was not strong, but comparable to previous studies
	Aguilar-Farías et al. (2014)	n = 37	Objective	The results suggest that cut-points are dependent on unit of analyses (i.e. epoch length and axes); cut-points for a given epoch length and axis cannot simply be extrapolated to other epoch lengths
	Hekler et al. (2012)	n = 870	Both	CHAMPS items effectively measured high-light, total activity, and MVPA in seniors, but further refinement is needed for sedentary and low-light activity
	van Uffelen et al. (2011)	n = 55	Subjective	The accuracy of older adults’ self-reported sitting time is questionable given the challenges they have in answering sitting-time questions
	Gardiner et al. (2011a)	n = 48	Both	The summary measure of total sedentary time has good repeatability and modest validity and is sufficiently responsive to change suggesting that it is suitable for use in interventions with older adults
Prevalence and types of SB	Shiroma et al. (2013)	n = 7247	Objective	Older women spent about two-thirds of waking time in SB, most of which occurred in bouts lasting less than 30 min
	Arnardottir et al. (2013)	n = 579	Objective	Sedentary time is high in Icelandic older adults who have high life-expectancy and live north of 60° northern latitude, while PA declines with increasing age and body mass index. Women spend more time in low-light PA, but less in MVPA than men
	Evenson et al. (2014)	n = 760	Objective	The New York sample spent a longer proportion of time in SB and light activities, but more time in MVPA than the country sample. Urbanicity may explain these differences
	Evenson et al. (2012)	n = 2630	Objective	MVPA estimates vary among adults aged 60 or older, depending on the cut point chosen, and most of their time is spent in SBs
	Lord et al. (2011)	n = 56	Objective	Walking, sedentary and transitory behaviours are distinct from each other, and together explain daily function
	Jefferis et al. (2015a)	n = 1419	Objective	Among older adults, the steep decline in total PA occurred due to reductions in MVPA whilst light PA is relatively spared and sedentary time and long sedentary bouts increase
Health impact of SB—Musculoskeletal health & functional fitness	Mitchell et al. (2015)	n = 5681	Subjective	SB was identified as mediator for the association between obesity and falls in community living older people
	Gianoudis et al. (2015)	n = 162	Subjective	Higher levels of SB in older adults were associated with reduced muscle mass and an increased risk of sarcopenia in community-dwelling older adults, independent of PA
	Dunlop et al. (2015)	n = 2286	Objective	These U.S. national data show a strong relationship between greater time spent in SB and the presence of ADL disability, independent of time spent in moderate or vigorous activity
	Santos et al. (2012)	n = 312	Objective	Elderly who spend more time in PA or less time in SBs exhibit improved functional fitness and other confounders
	Chastin et al. (2012)	n = 30	Objective	The pattern of SB accumulation varies between older adults and is associated with muscle quality and adiposity
	Cawthon et al. (2013)	n = 1983	Objective	Older men with lower total energy expenditure, lower moderate activity, or greater sedentary time were more likely to develop a functional limitation
Health impact of SB—Cardio metabolic health & mortality	Ensrud et al. (2014)	n = 2918	Objective	In older men exceeding current guidelines on PA, greater time spent in SB is associated with increased mortality risk
	Chase et al. (2014)	n = 54	Objective	SB is associated with an adverse metabolic effect on low-density lipoprotein in seniors, even those who meet guideline recommendations for an active ‘fit’ adult
	Gennuso et al. (2013)	n = 1914	Objective	The results suggest that sufficient MVPA did not ameliorate the negative associations between SB and cardio metabolic risk factors or functional limitations in the current sample
	Inoue et al. (2012)	n = 1806	Subjective	Spending less time watching TV, a predominant SB, was associated with lower risk of being overweight or obese, independent of meeting PA guidelines
	Stamatakis et al. (2012)	n = 2765	Both	SB is associated with cardio metabolic risk factors, but the associations are more consistent when it is measured by self-report that includes TV viewing
	Gardiner et al. (2011c)	n = 1958	Subjective	High levels of SB were associated with greater prevalence of the metabolic syndrome
	Bankoski et al. (2011)	n = 1367	Objective	The proportion of sedentary time was strongly related to metabolic risk, independent of PA
	Gao et al. (2007)	n = 455	Subjective	A high prevalence of the metabolic syndrome in a representative sample of Caribbean-origin Hispanic elders was associated with prolonged television viewing, independent of PA and energy intake
	León-Muñoz et al. (2013)	n = 2635	Subjective	Compared with consistently sedentary older adults, consistently non-sedentary individuals showed reduced all-cause mortality. Individuals who changed sitting time experienced an intermediate reduction in mortality
	Pavey et al. (2015)	n = 6656	Subjective	Prolonged sitting-time was positively associated with all-cause mortality. Women who reported sitting for more than 8 h/day and did not meet PA guidelines had an increased risk of dying within the next 9 years
	Gómez-Cabello et al. (2012)	n = 3136	Subjective	Sitting time increases the risk of overweight-obesity and overfat in women and the risk of central obesity in men, independently of walking time
Health impact of SB—Other (health) outcomes & quality of life	Withall et al. (2014)	n = 228	Objective	Steps, MVPA and lower limb function were independently and moderately positively associated with perceived physical well-being but relationships with mental well-being variables were weak. No significant associations between SBs and well-being were observed
	Balboa-Castillo et al. (2011)	n = 1097	Subjective	Greater leisure-time PA and less leisure-time SB were independently associated with better long-term health-related QoL in older adults
	Vance et al. (2008)	n = 158	Subjective	Partial support was found for PA to improve and SB to worsen cognitive health
	Verghese et al. (2003)	n = 469	Subjective	Participation in certain seated leisure activities (like reading or playing board games) is associated with a reduced risk of dementia, even after adjustment for base-line cognitive status and after the exclusion of subjects with possible preclinical dementia
Strategies to counteract the health effects of SB	Meneguci et al. (2015)	n = 3296	Subjective	Socio-demographic, clinical, and health behaviour factors are associated with high sitting time in older adults from South-eastern Brazil
	Sardinha et al. (2015)	n = 215	Objective	Breaking-up sedentary time is associated with better physical function in older adults; and, it may have an important place in future guidelines on preserving older adults’ physical function to support ADL
	Gardner et al. (2014)	n = 120	Both	N/a
	Chastin et al. (2014b)	n = 11	Subjective	Older adults consider self-efficacy, functional limitations, ageist stereotyping, locus of control, and pain as determinants of their SB
	van der Berg et al. (2014)	n = 565	Objective	Some demographic, socioeconomic, and biomedical determinants in midlife were associated with considerably more sedentary time per day in old age
	Van Cauwenberg et al. (2014a)	n = 50,986	Subjective	There is a cross-sectional link between older adults’ television viewing time and social composition of their neighbourhood, formal participation, access to alternative activities, and safety from crime
	Fitzsimons et al. (2013)	n = 24	Both	A consultation approach may help individuals reduce time spent in SBs
	Davis et al. (2014)	n = 217	Objective	Promoting regular breaks in sedentary time might be useful in maintaining or increasing lower extremity function and later life independence
	Kikuchi et al. (2013)	n = 1665	Subjective	Particular socio-demographic and behavioural characteristics related to TV time among Japanese older adults have been identified, but they differ by gender
	Gardiner et al. (2011b)	n = 59	Objective	Sedentary time in older adults can be reduced following a brief intervention based on goal setting and behavioural self-monitoring
	Nicklas et al. (2014)	n = 48	Objective	Self-monitoring of spontaneous PA and SB enhanced successful maintenance of lost weight
	Uffelen et al. (2012)	n = 6116	Subjective	It is suggested that older women with a high health risk profile and social risk profile may particularly benefit from interventions to promote both reducing sitting time and increasing PA or at least light activities
	Dogra and Stathokostas (2014)	n = 14,560	Subjective	Several specific correlates of extended sitting time were identified; these findings have implications for public health strategies targeting older adults

Data presented in paragraph(s)	Author(s)	Study population	Subjective or objective SB tool	General finding(s)
Reviews				
Prevalence and types of SB	Harvey et al. (2013)	n = 372,550	Both	Whether measurements are subjective or objective, the majority of older adults are sedentary
	Harvey et al. (2015)	n = 349,698	Both	Time spent sedentary ranges from 5.3 to 9.4 h per waking day in older adults
Health impact of SB—Overall	de Rezende et al. (2014a)	n = 335,503	Both	The data supports the relationship between SB and mortality in older adults

*SB* sedentary behaviour, *CHAMPS* community healthy activities model program for seniors, *MVPA* moderate-to-vigorous physical activity, *PA* physical activity, *ADL* activities of daily living, *TV* television, *QoL* quality of life, *N/a* not applicable

## Assessment of sedentary behaviour

Similar to characterising PA and exercise by the FITT formula, describing the Frequency, Intensity, Time (duration) and Type of activity, SB is suggested to be characterised by the SITT formula, which describes Sedentary behaviour frequency, number of Interruptions, Time (duration) and Type (Tremblay et al. 2010). These variables provide valuable information on SB and should therefore be assessed in any study dealing with SB. Since the need to quantify SB emerged, efforts have been undertaken to develop suitable measurement techniques. Overall, these can be classified as either subjective or objective, and both have different outcome measures. According to previous research (Pate et al. 2008; Chastin and Granat 2010; Pedišić and Bauman 2015), studies on SB initially relied on self-reported methods, such as questionnaires and/or logs. Subjective methods are practical, easy to administer, inexpensive, useful in large-scale studies and do not alter behaviour (Celis-Morales et al. 2012; Chastin et al. 2014a; Aguilar-Farías et al. 2015). They will provide SB outcomes in terms of total sitting time, total screen time or TV time. If surrogate or proxy SB measures (e.g. TV viewing or total screen time) are used as an indicator of total SB, conclusions can however only be drawn limited to the used measures, because the association with total objective SB seems rather weak, even if the proxy measure is objective (Pate et al. 2008; Visser and Koster 2013; Chastin et al. 2014a). Although the number of SB questionnaires for older adults increases and quality improves in terms of acceptable reliability measures, validity of self-reported total sedentary time against accelerometer-derived SB is not strong yet (Gardiner et al. 2011a; Hekler et al. 2012; Visser and Koster 2013; Van Cauwenberg et al. 2014b; Aguilar-Farías et al. 2015). A major flaw is that most studies validate questionnaires against sensors unable to capture SB accurately due to the inability of measuring postural orientation, e.g. thigh inclination (Chastin et al. 2014a). Generally, most subjective measures have obvious caveats, like bias and the tendency to under-report SB (Chastin and Granat 2010; Harvey et al. 2015; Aguilar-Farías et al. 2015). SB appears to be more difficult to recall than PA, because of its habitual nature (Hart et al. 2011; Bond et al. 2014). Especially for older adults it is a challenge to accurately estimate sitting-time (van Uffelen et al. 2011). The combination of underestimation and low precision is likely to reduce the ability to accurately detect dose–response relationships between self-reported SB and health outcomes (Chastin et al. 2014a). Nevertheless, so-called past or previous day recall questionnaires have been reported as promising since they are easy-to-administer, compare favourably with other sedentary time questionnaires, criterion validity is high, and systematic errors low (Clark et al. 2013; Matthews et al. 2013). Self-reports might give a detailed picture of how, where and why SB time is spent, which could be essential for developing interventions and public policy (Rhodes et al. 2012; Matthews et al. 2013; Kozey Keadle et al. 2014; Van Cauwenberg et al. 2014b; Busschaert et al. 2015). Thus, subjective methods can provide useful information and should not be ignored in SB assessment, but they should not be used as sole means to assess SB, and the development of accurate self-report tools to measure (specific) SB in elderly is still required (Van Cauwenberg et al. 2014b; Gennuso et al. 2015).

Although many objective techniques are available to capture PA, there are only few to measure SB, in particular accelerometers (Tremblay et al. 2010). Accelerometry is preferred by most studies since it provides reliable and valid measures of both PA and SB, and it overcomes many of the above-mentioned limitations of self-reports (Evenson et al. 2012; Gorman et al. 2014; Lohne-Seiler et al. 2014; Aguilar-Farías et al. 2014; Pedišić and Bauman 2015). However, it is important to mention that different accelerometers use distinct methods to measure SB. One quantifies SB by a lack of movement, and the other by postural allocation. The first type only uses estimates of energy expenditure in combination with cut-off points to define SB. However this results in misclassification as standing is difficult to distinguish from sitting when performed below the sedentary cut-off point (Stamatakis et al. 2012; Aguilar-Farías et al. 2014). Devices measuring postural allocation are more accurate in assessing SB and therefore not only recommended but also used as reference standard (Kozey-Keadle et al. 2011; Aguilar-Farías et al. 2014). When compared to self-reports, accelerometers are expensive (≥£190 per unit), there is potential bias due to a Hawthorne effect (behaviour change in response to the awareness of being observed) and data-analysis is labour-intensive (Visser and Koster 2013; Pedišić and Bauman 2015), at least until an analysis template has been created. However, accelerometry enables more robust, objective, ambulatory and long-term recording of acceleration signals (Chastin and Granat 2010; Tremblay et al. 2010), and provides outcomes, such as total SB time, sedentary bout time, sedentary pattern, and number and frequency of breaks in SB. Nonetheless, accelerometry only addresses the energetic ontology of the definition of SB and there is no consensus on a standardised method for accelerometer data processing and analysis (e.g. non-validated cut-points or epoch lengths) (Gorman et al. 2014; Pedišić and Bauman 2015). Assumptions are still required to quantify accelerometry-based PA and SB in older adults, resulting in a potential danger of misinterpretation (Evenson et al. 2012; Kowalski et al. 2012; Gorman et al. 2014; Kozey Keadle et al. 2014). With modern technological advances, accelerometer use is assumed to be more straightforward and easy to implement. Furthermore, the possibilities of objective SB monitoring will continue to increase and provide an ever more-detailed and accurate objective picture of SB in elderly.

The main reason for preferring accelerometry in SB measurement is that it provides an objective assessment of SB and may thereby help to understand how SB is related to healthy ageing (Visser and Koster 2013; Van Cauwenberg et al. 2014b). Nevertheless, accelerometers should not substitute but supplement questionnaires (Pedišić and Bauman 2015). Self-reports are still needed to assess engagement in specific SBs and provide more detailed (qualitative) information that cannot be obtained with accelerometers (Rhodes et al. 2012; Lohne-Seiler et al. 2014; Van Cauwenberg et al. 2014b). Generally, it is suggested that SB associations are complex to interpret because they depend on the type of SB studied and the measurement method used (Table 2) (Stamatakis et al. 2012; de Rezende et al. 2014b). For example, Lenz (2014) noted that in older adults TV viewing had more associations with cardio metabolic outcomes than reports of total SB, while Celis-Morales et al. (2012) concluded that, due to underestimation, self-reports might miss some significant trends that will be found when objective assessments are used.

**Table 2:** Brief overview of the complex SB associations with cardio metabolic outcomes

SB associations	Adults (18–73 years)		Older adults (≥60 years)													
	Celis-Morales et al. (2012) (n = 317)		Gennuso et al. (2013) (n = 1914)	Lenz (2014) (n = 70)						Gao et al. (2007) (n = 455)	Gardiner et al. (2011c) (n = 1958)		Stamatakis et al. (2012) (n = 2765)			
	Obj.	Subj.	Obj.	Subj.						Subj.	Subj.		Obj.	Subj.		
Cardio metabolic risk factors	Acc.	Total SB	Acc.	TV	Reading	Eating	Computer	Transport	Total SB	TV	TV	Total SB	Acc.	TV	Non- TV leisure sitting	Total SB
Glucose/GI	**+**	**+**	**+**		**+**						**+**					
Insulin	**+**	**+**														
HOMA_IR_/diabetes	**+**	**+**												**+**	**+**	**+**
HbA1C																
TC/CR	**+**	**+**								**+**				**+**		**+**
HDL	**+**	**+**		**+**					**+**	**+**	**+**	**+**				
LDL	**+**	**+**														
TG	**+**	**+**		**+**								**+**				
SBP	**+**	**+**		**+**						**+**		**+**				
DBP	**+**	**+**								**+**		**+**				
Weight			**+**						**+**							
BMI/overweight/Obesity	**+**	**+**	**+**							**+**				**+**		**+**
WC/WHR/AO	**+**	**+**	**+**	**+**					**+**	**+**		**+**	**+**	**+**		**+**
Body fat	**+**	**+**		**+**		**+**										
CRP			**+**													

*SB* sedentary behaviour, *Obj.* objective method, *Subj*. subjective method, *Acc*. accelerometer, *TV* television viewing, *GI* glucose intolerance, *HOMA*_*IR*_ homeostasis model assessment-estimated insulin resistance, *TC* total cholesterol, *CR* cholesterol ratio, *HDL* high-density lipoprotein, *LDL* low-density lipoprotein, *TG* triglycerides, *SBP* systolic blood pressure, *DBP* diastolic blood pressure, *BMI* body mass index, *WC* waist circumference, *WHR* waist-to-hip ratio, *AO* abdominal obesity, *CRP* C-reactive protein, *HbA1C* glycated haemoglobin, + significant association

When capturing SB in older adults, different parameters have to be taken into account, depending on the method applied, i.e. mounting position, data filtering and algorithm, and type of device and/or questionnaire used. Additionally, potential confounders like age, gender, health status or socioeconomic status have to be considered. Another important consideration to accurately estimate SB is the number of complete data acquisition days needed. Compared with PA, more monitoring days are needed to reliably estimate SB because it is less predictable on a daily basis (Hart et al. 2011). In older adults, 5 monitoring days are required to provide a reliable (ICC = 0.80) SB estimate when using an objective method, while only 3 days are necessary to monitor PA with the same level of reliability (Hart et al. 2011). Increasing the number of monitoring days to either 7, 11 or 21, will improve the reliability of SB monitoring resulting in ICCs of 0.85, 0.90 and 0.95 respectively (Hart et al. 2011). Since studies are divergent on whether there is a difference in SB between week and weekend days in older adults, it is advised to include both when using a <7-day monitoring protocol (Hart et al. 2011; Davis et al. 2011; Visser and Koster 2013). Compared to objective methods, self-reports show larger day-to-day differences and therefore they require more monitoring days (preferably ≥7) to reliably predict SB (Hart et al. 2011).

Generally, SB assessment in older adults is challenging, regardless of the method applied or outcome measures used. A combination of both objective (using postural allocation) and self-reported methods used in a 7-day monitoring protocol is currently suggested to be optimal for assessing SB in older adults.

## Prevalence and types of sedentary behaviour

Daily function in older adults is mainly subdivided in walking, postural transitions and SB (Lord et al. 2011), with several studies reporting that most of their time is spent in SBs (Healy et al. 2008; Davis et al. 2011; Evenson et al. 2012; Shiroma et al. 2013; Jefferis et al. 2015b). Previous literature shows that SB increases with age, resulting in older adults (aged ≥60 years) being the most sedentary (Matthews et al. 2008; Rhodes et al. 2012; Martin et al. 2014) and old-older adults being more sedentary than young-older adults (Table 3) (Evenson et al. 2012; Martin et al. 2014; Harvey et al. 2015). Interestingly, after retirement (from ~65 years of age) the amount of SB transiently reduces, while the percentage of ambulatory activity increases (Godfrey et al. 2014). Not only the amount of SB and long sedentary bouts increase with ageing in older adults, but also the decline in total daily PA accelerates (Table 3) (Davis et al. 2011; Harvey et al. 2013; Buchman et al. 2014; Martin et al. 2014; Jefferis et al. 2015a). This latter decline is characterized by: (1) lower PA volume, (2) less higher-intensity PA, and (3) lower frequency of getting out and about (Davis et al. 2011). This results in old-older adults (aged ≥85 years) performing only one third of the activity performed by young-older adults (aged 70–74.9 years) at peak activity times (Davis et al. 2011).

**Table 3:** Comparison of accelerometer-derived SB across different age groups

Matthews et al. (2008)							
	Age groups						
	16–19	20–29	30–39	40–49	50–59	60–69	70–85
Male	7.9	7.3	7.2	7.6	7.9	8.8	9.5
Female	8.1	7.7	7.3	7.5	7.8	8.1	9.1

Martin et al. (2014)				
	Age groups			
	20–39	40–59	60–69	≥70
Male	7.9	8.5	9.4	10.3
Female	7.9	8.3	8.7	9.8

Values represent mean hours/day adjusted for monitor-wearing time

*SB* sedentary behaviour

According to national surveys, adults are on average sedentary for 8 h of the waking day, and this figure rises to >10 h in older adults (Matthews et al. 2008; Davis et al. 2011; Lenz 2014). However, two systematic reviews describe that self-reported SB in older adults (aged ≥60 years) is on average 5.3 h/day only (Harvey et al. 2015), with ~60 % reporting sitting >4 h/day during waking hours (Harvey et al. 2013). When using objective measurements, older adults (aged ≥60 years) spend on average 8.5–9.6 h/day sedentary (Evenson et al. 2012, 2014; Harvey et al. 2015), which equals 65–80 % of their waking day. Other accelerometer-based studies showed that older adults spend approximately 75–80 % of their awake time in SB which represents 8–12 h/day (Arnardottir et al. 2013; de Rezende et al. 2014a). Other studies suggest that 67 % of the older age population is sedentary for >8.5 h/day (Stamatakis et al. 2012), and that about half (47 %) of them are sedentary >80 % of their waking hours (Davis et al. 2011). In general, older adult men spend more time in SB (~75 % of the day) than older adult women (~66 % of the day), but in both the total time of SB is primarily the result of accumulation of many relatively short SB bouts of less than 30 min (Davis et al. 2011; Evenson et al. 2012; Shiroma et al. 2013; Harvey et al. 2015; Jefferis et al. 2015b).

For a better and more detailed understanding of SB, it is important to assess typical SBs. Previous research has shown that older adults engage in approximately 16 types of SB daily, with TV viewing, reading, eating meals, computer use and transportation being the most common (Lenz 2014). Generally, TV viewing and computer use are the main SB measures, followed by the overall assessment of time-spent sitting (van Uffelen et al. 2011; Rhodes et al. 2012; Visser and Koster 2013). Time spent TV viewing combined with computer use is termed screen time (Harvey et al. 2013). About 53 % of the older adults report daily screen time >4 h, and ~94 % >2 h (Harvey et al. 2013). When splitting daily screen time, older adults watch on average 3.3 h TV, with more than half of the age group (54 %) sitting in front of the TV for 3 h, while about one third watches TV >3.6 h and 15 % >4 h daily (Harvey et al. 2013). Around 65 % of older adults use computers, but <10 % use it more than 1.6 h daily (Harvey et al. 2013). A more general outcome, like leisure sitting time (excluding TV time), is reported by older adults to be on average 3.3 h daily, and reported by ~54 % to be >3 h (Patel et al. 2010; Harvey et al. 2015). Total sitting time >3 h is reported in older adults by 78 %, with ~59 % reporting sitting >4 h, ~27 % reporting >6 h and 5 % reporting >10 h daily (Harvey et al. 2013).

Although the amount of SB varies in the current literature depending on the assessment method used (range 5.3–12 h/day), it is nevertheless clear that SB is highly prevalent in older adults. PA appears to be lower and of less intensity, making light-intensity PA (LIPA) the most common type of PA within the oldest age groups (Table 3). This suggests that LIPA is the most feasible PA in elderly, which is of interest to counteract SB, as will be discussed later.

## Sedentary physiology

Research into the physiology and health impacts of SB has recently increased and represents an exciting new field of study, which is distinct but complementary to exercise physiology, namely sedentary physiology (Tremblay et al. 2010; Sedentary Behaviour Research Network 2012; Dunstan et al. 2012a). Associations between SB and several outcomes have been reported. However, the mechanisms underlying the association between SB and adverse health effects remain uncertain and are therefore a research priority (Dunstan et al. 2012a; Gianoudis et al. 2015). To date, physiological mechanisms for four different outcomes have been proposed regardless of age, namely:

- *Cardio metabolic* It has been proposed that reduced energy expenditure and muscle contractions not only lead to reduced insulin sensitivity and an increase in pro-inflammatory cytokines (Tremblay et al. 2010; Yates et al. 2012), but also decreased lipoprotein lipase (LPL) activity and muscle glucose transporter (GLUT) protein content (Tremblay et al. 2010; Gianoudis et al. 2015);
- *Vascular* Studies have shown that shear rate, FMD and brachial artery diameter decrease, while endothelial cell damage and blood pressure increase with increasing SB (Demiot et al. 2007; Hamburg et al. 2007; Thosar et al. 2015);
- *Muscle–tendon* It is proposed that continual under-loading due to SB, negatively affects muscle–tendon properties, since muscle–tendon disuse causes changes (e.g. muscle atrophy and increased tendon compliance). Aside from that, SB is thought to be a determinant driver for obesity (Chastin et al. 2012). Generally, it is proposed that an increase in visceral and intermuscular fat stimulates the release of pro-inflammatory cytokines and decrease of anti-inflammatory markers from adipose tissue, having a catabolic effect on muscle tissue by impairing muscle protein synthesis (Gianoudis et al. 2015). This will affect muscle performance, however that does not only arise from muscular but also neural factors (Tomlinson et al. 2014);
- *Skeletal* SB is thought to change the balance between bone resorption and deposition, mainly by a rapid increase in bone resorption (marked by increased deoxypyridinoline, urinary calcium and type I collagen cross-linked N-telopeptides) without concomitant changes in bone formation, resulting in reduced bone mineral content and increased risk of osteoporosis (Kim et al. 2003; Tremblay et al. 2010).

## Health impact of sedentary behaviour

**Fig. 2:**
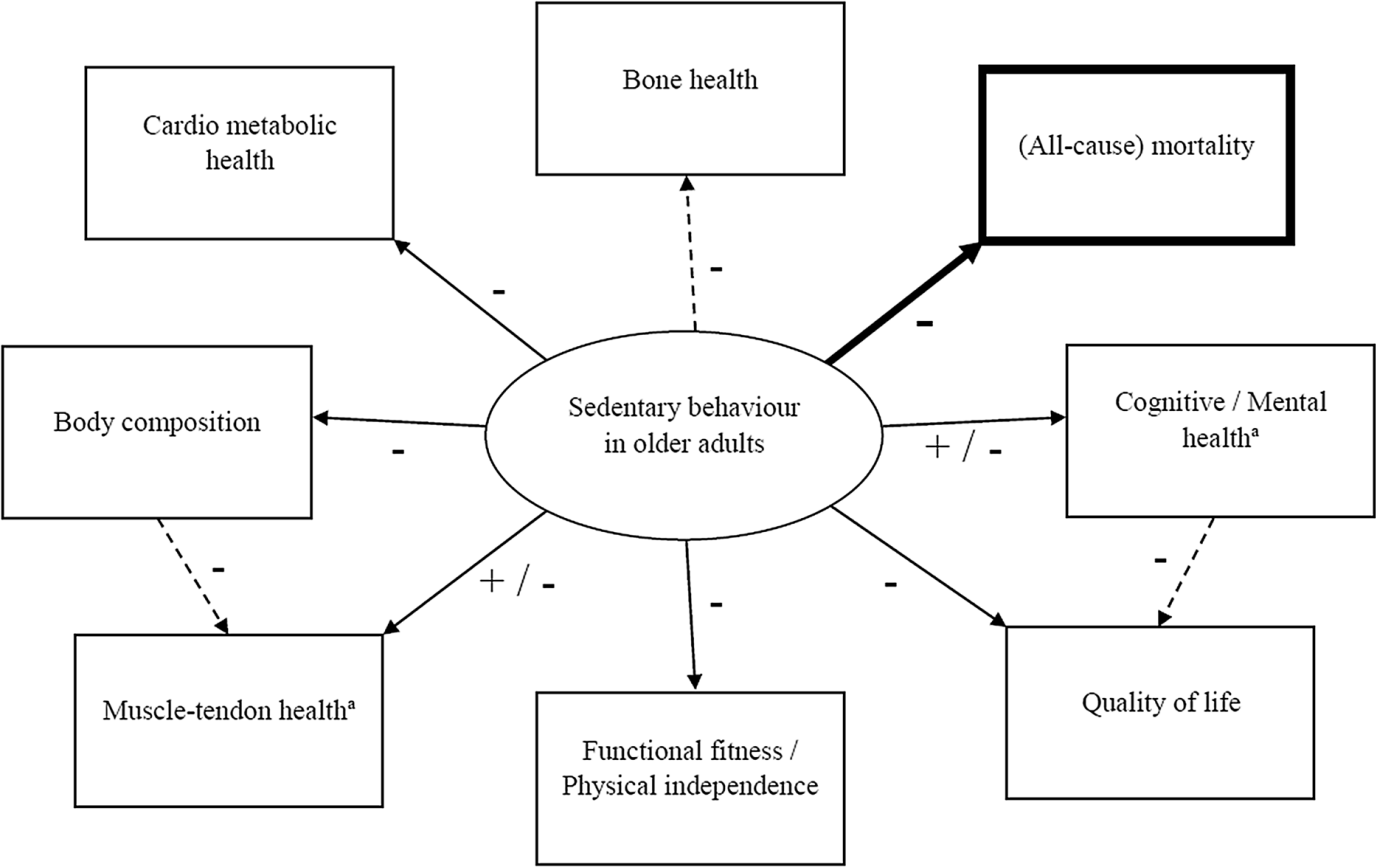
Overview of identified and suggested associations between SB and (health) outcomes in older adults as reported in literature + positive association; − negative association; *solid lines* represent identified associations; *dashed lines* represent suggested associations; Associations in *bold* are confirmed by a systematic review from de Rezende et al. (2014a). ^a^Outcome depends on the type of assessed SB (e.g. TV viewing, computer use or reading)

Despite a high prevalence, SB in older adults has so far received limited scientific attention (Gennuso et al. 2013; Van Cauwenberg et al. 2014b). A general overview of reported (health) outcomes, independently associated with SB in healthy, community-dwelling older adults, is provided below (Fig. 2).

### Musculoskeletal health & functional fitness

Although proof of SB effects on musculoskeletal health is limited in elderly, some interesting findings have been reported. Evidence shows for example, that associations between screen-based SB and muscle strength, independently of PA, are context-specific where TV viewing is associated with lower muscle strength while opposite effects are observed for computer use (Hamer and Stamatakis 2013). This might result from lower energy expenditure and unhealthier eating behaviours during TV watching, but also a potential confounding effect of education level on computer use (Visser and Koster 2013; Lenz 2014). Further, a study examining the relation between sarcopenia and SB, showed that higher volumes of TV viewing time were related to lower total body and leg lean mass after adjusting for fat mass, which was positively associated with the duration of watching TV (Gianoudis et al. 2015). Another study confirmed this latter finding by suggesting that SB is directly related to (lower limb) adiposity in older men, but increased and prolonged SB was also, unexpectedly, associated with increased leg power and muscle quality in these men (Chastin et al. 2012). Possible explanations for this latter finding were, e.g. carrying more body fat may provide a training stimulus or results reflect adiposity developing in previously strong men who have recently become sedentary. However, according to Chastin et al. (2012), their results should be interpreted with caution since the study sample was not necessarily representative of elderly in general. Other research shows that higher levels of SB in older adults are associated with an increased risk of sarcopenia and limited physical function, independent of PA or other potential confounding factors (Gennuso et al. 2013; Gianoudis et al. 2015). These findings are confirmed by other studies showing that even after adjusting for PA and other confounders, objectively measured SB is negatively associated with functional fitness and the ability to perform activities of daily living (Santos et al. 2012; Cawthon et al. 2013; Dunlop et al. 2015). According to Marques et al. (2014), SB is only a predictor for the risk of losing physical independence when not controlling for PA intensities. However, this finding might result from misclassification of participants due to using accelerometer data of less than five monitoring days and a self-reported measure of physical function. Santos et al. (2012) found that PA was positively related to functional fitness, independent of SB, and therefore they concluded that both SB reduction and PA increase in older adults might preserve functional fitness and performance in terms of daily functioning tasks and independent living. Especially obese people could benefit from this since SB has been identified as a mediator for the association between obesity and falls in elderly (Mitchell et al. 2015). A study on successful ageing, which represents the physical, psychosocial, and social success with which adults age, showed that SB is associated with lower odds of successful ageing (Dogra and Stathokostas 2012). Although a dose-dependent relationship exists between SB and each of the three successful ageing components, the strongest association was found between SB and functional limitations (physical component) (Dogra and Stathokostas 2012). Functional dependence in old age is more likely to develop in older adults who are not physically active, or who were not so during their middle age (Dogra and Stathokostas 2012; Marques et al. 2014).

Skeletal measures are limited to a single report, showing that independent of time spent engaging in PA, SB is negatively associated with femur bone mineral density in older women only (Chastin et al. 2014c).

### Cardio metabolic health & mortality

Regarding risk factors for cardio metabolic diseases, TV viewing and self-reported SB are positively associated with (i) dyslipidaemia characterised by increased triglycerides and lower high-density lipoprotein (HDL), (ii) obesity, (iii) hypertension and (iv) glucose intolerance (in women only) (Gao et al. 2007; Gardiner et al. 2011c; Inoue et al. 2012; Lenz 2014). These findings are in agreement with another study suggesting that self-reported SB (TV viewing in particular) and, to a lesser extent, objectively measured SB in older adults are negatively associated with two cardio metabolic risk proxies, independently of PA: (1) cholesterol index and (2) diabetes prevalence (Stamatakis et al. 2012). Gennuso et al. (2013) also reported that associations between accelerometer-derived SB and various health outcomes in older adults were not modified by PA, however they only found independent associations with body mass (index), waist circumference, C-reactive protein and plasma glucose, but not with blood pressure, cholesterol markers and triglycerides (Gennuso et al. 2013). Nevertheless, Chase et al. (2014) showed that objectively measured SB was associated with an adverse metabolic effect on low-density lipoprotein (LDL) levels in physically active elderly. Overall, most studies suggest that watching TV and/or engaging in large amounts of total SB is negatively associated with the (cardio metabolic) health of older adults (Bankoski et al. 2011; Gardiner et al. 2011c; Gómez-Cabello et al. 2012; Lenz 2014). Moreover, SB also negatively affects mortality independently of PA, either or not caused by cardio metabolic disorders (Dogra and Stathokostas 2012; Stamatakis et al. 2012; Martínez-Gómez et al. 2013; León-Muñoz et al. 2013; Ensrud et al. 2014; Pavey et al. 2015).

### Other (health) outcomes & quality of life (QoL)

Although Withall et al. (2014) did not find an association between SB and subjective well-being of older adults, evidence shows that in the elderly, less leisure-time SB is independently associated with better long-term health-related QoL and cognitive performance (Balboa-Castillo et al. 2011; Steinberg et al. 2015). The number of sitting hours were inversely related with the scale scores of physical functioning, physical role, bodily pain, vitality, social functioning and mental health (Balboa-Castillo et al. 2011). Obesity, diabetes and hypertension are possible mediating mechanisms for these associations between SB and well-being (Balboa-Castillo et al. 2011). As stated earlier in this review, leisure-time SB types are differently associated with health markers in older adults (Kesse-Guyot et al. 2012; Kikuchi et al. 2014). For example, higher passive SB (e.g. TV viewing) is associated with a higher likelihood of being overweight, adverse health behaviours (like poor diet) and greater psychological distress, while mentally-active sedentary time (i.e. reading or computer use) is not associated with health-related attributes and may involve (i) beneficial processes which prevent for the deleterious impact of sitting in older adults, (ii) provide mental stimulation improving cognitive performance capacities and (iii) improve social interaction and QoL (Verghese et al. 2003; Vance et al. 2008; Kesse-Guyot et al. 2012; Visser and Koster 2013; Kikuchi et al. 2014). Overall across age groups, most sedentary activities are suggested to decrease communication with family, reduce the social network and increase the risk of depression, anxiety and stress, which would explain the poorer QoL associated with SB (Balboa-Castillo et al. 2011).

In spite of the limited number of SB studies in older adults, evidence is growing on the (in general) adverse health effects of SB. A recent systematic review by de Rezende et al. (2014a), accounting for the quality of SB studies in older adults (assessed with the Grades of Recommendation, Assessment, Development and Evaluation (GRADE) tool), suggests, however, that to date evidence is inconclusive. Due to the limited quality of available studies, only scarce evidence exists for all the reported health outcomes associated with SB in elderly, except for the evidence on a previously established dose–response relationship between SB and all-cause mortality, which was confirmed (Fig. 2) (de Rezende et al. 2014a). Moreover, the evidence on musculoskeletal health and functional fitness in relation to SB in elderly, has not been graded by de Rezende et al. (2014a). Overall, the present evidence of independent associations between SB and health outcomes in older adults should be carefully interpreted, and further research, to either support or refute the current findings, is needed to draw firm conclusions which will lead to informed SB-minimisation strategies and guidelines for older adults (de Rezende et al. 2014a).

## Strategies to counteract the health effects of sedentary behaviour

Regardless of the inconclusive evidence on all of the possible negative health effects of SB in older adults, multiple studies have already proposed strategies to counteract the health impact of SB. These strategies can be classified as either interventional or preventative.

Generally, research shows that especially prolonged sedentary bouts instead of frequent sedentary bouts, have negative health effects, and therefore sitting duration should be focused on more than on frequency (Bond et al. 2014; Chastin et al. 2014c). To date, several studies on different age groups (including older adults) have already shown that breaking prolonged sedentary bouts can be effective, particularly in decreasing the cardio metabolic disease risk (Healy et al. 2008; Bankoski et al. 2011; Bond et al. 2014; Gianoudis et al. 2015; Bailey and Locke 2015), while results on musculoskeletal health and function appear to be equivocal (Gianoudis et al. 2015). Nevertheless, both Sardinha et al. (2015) and Davis et al. (2014) found an association between breaks in SB and better physical function in older adults. Although all these findings make breaking prolonged SB a very promising intervention, it has not been studied as such in elderly yet and, only few studies have been conducted to promote adoption of this approach overall (Bond et al. 2014). In general, it is not necessary to decrease SB dramatically before any health effect can be achieved. This was shown by Pronk et al. (2012), who noted that only 16 % decrease in SB already generated health benefits in employees with sedentary jobs. Other non-elderly studies reported improved cardio metabolic factors in participants breaking every 20–30 min of sitting with just ~2 min of PA (Dunstan et al. 2012b; Peddie et al. 2013; Bailey and Locke 2015). These results are highly stimulating in counteracting SB, since it is a habitual lifestyle and therefore difficult to change (Hart et al. 2011; Bond et al. 2014).

It appears that the intensity of the SB interruption is an important factor regarding its health effect (Chastin et al. 2012; Bailey and Locke 2015). Bailey and Locke (2015) showed that interrupting sitting with standing alone is not sufficient and that at least LIPA (e.g. light-intense walking) is required. A possible explanation is that minor increases in contractile activity (which are associated and easily achieved with LIPA) can dramatically increase muscle GLUT-1 & 4 content and glucose tolerance in sedentary individuals (Tremblay et al. 2010; Latouche et al. 2013; Sardinha et al. 2015). This is ideal, since LIPA is not only inversely related with SB, but also a feasible approach for older adults to increase total PA and ameliorate the deleterious health effects of SB (Hamilton et al. 2008; Healy et al. 2011). However, it needs to be determined if there might be any adverse consequences of shifting SB into LIPA, especially in case of older adults who may be more prone to lower-body musculoskeletal problems (Tremblay et al. 2010). Changing SB to moderate-to-vigorous PA (MVPA) (e.g. brisk walking, walking stairs or exercising) would potentially lead to spontaneous compensatory behaviour resulting in a less fragmented and possibly, higher total SB in turn, and is therefore not preferred (Chastin et al. 2012). Epidemiologic evidence suggests that having a positive balance between LIPA and SB is desirable due to the inverse linearity of LIPA with a number of cardio metabolic biomarkers (Hamilton et al. 2008). It is known that physiological responses and adaptations may differ within and between physiological systems (Tremblay et al. 2010). For sedentary people it is suggested that LIPA might only have beneficial effects on the cardiovascular and metabolic systems, but not on the musculoskeletal system possibly due to a lack of overload, which is normally required for improvement of this particular system. Results from a preliminary study support this and suggest that vigorous PA during breaks is associated with higher muscle quality in older adults (Chastin et al. 2012). However, new evidence from a small study in young males (mildly active only i.e. not involved in any type of exercise program and not having undergone a systematic resistance training program within 1 year prior onset of the intervention) indicates that also mild walking can improve muscle strength (Maeo et al. 2015). Nevertheless, small changes from SB to LIPA can already lead to a decrease in risk for chronic diseases and mortality (Tremblay et al. 2010). Moreover, these small changes also increase physical functioning which reduces the risk of falls, allowing older adults to live independently and enhance the quality of later life (Sardinha et al. 2015). These advantages are not necessarily associated with MVPA and do also not require prolonged periods of PA (Sardinha et al. 2015). However, regular MVPA is still important in the prevention and treatment of chronic diseases, even in older adults (Dunstan et al. 2012a). Therefore, both PA and SB should be part of general guidelines, but more studies are needed to create informed guidelines for SB in the elderly (de Rezende et al. 2014a). In addition to breaking prolonged SB and reducing total SB, studies have also reported that specific, primarily passive SB (e.g. TV watching) should be targeted, since this type of SB is also related to other adverse health behaviours, like poor diet (Visser and Koster 2013). Overall, no definitive recommendations regarding the maximum total SB, number and duration of breaks, and optimal interventional strategy to stimulate breaking prolonged SB exists currently, as it requires more research (Dunstan et al. 2012a).

Regardless of this, as well as motivational interviewing (which was successful in stimulating PA in elderly (Letourneau and Goodman 2014), as the emerging use of technology might be promising tools to stimulate and alert breaks in SB. A recent example of the latter method is a study by Bond et al. (2014) who successfully used smartphone and activity monitor applications that provide personal feedback and prompt frequent short sitting breaks based on real-time data. However, their study was performed on a middle-aged population, so it is unclear whether this will also be effective in older adults, but expectations are high. Although interventions might be successful in the short-term, future research is necessary to examine also the long-term post-intervention effects on the amount and pattern of SB and PA. In order to design successful intervention programs it is important to know what reasons (apart from health or age) older adults might have that make them (more) sedentary or stay inside, such as social, economic and environmental factors (Uffelen et al. 2012; Kikuchi et al. 2013; Van Cauwenberg et al. 2014a; Dogra and Stathokostas 2014; Meneguci et al. 2015). A preliminary study by Chastin et al. (2014b) reported some specific factors, considered as determinants of SB by older adults themselves, like self-efficacy, functional limitations, ageist stereotyping, locus of control (the extent to which people believe they have personal control over events and outcomes in their lives), and pain. Considering these factors when designing SB-reducing interventions, might presumably lead to tailored strategies with high efficacy (Chastin et al. 2014b). Other characteristics of successful intervention programs to reduce SB in older adults might include personalised goal setting and feedback as part of behavioural self-monitoring using a consultation approach (Gardiner et al. 2011b; Fitzsimons et al. 2013). Something like this was already proven successful in preventing weight regain in elderly (Nicklas et al. 2014). Or maybe even some form of reinforcement or habit formation like in a newly ‘On Your Feet to Earn Your Seat’ randomized controlled trial (Gardner et al. 2014).

Instead of interventions, it might also be useful to see whether large amounts of (prolonged) SB can be prevented in elderly. Therefore, it is important to gain knowledge about the risk factors of SB. Previous research has shown that demographic, socioeconomic and biomedical variables in midlife (e.g. not being married, primary education, living in a duplex or living in an apartment (vs. villa), being obese, and having a heart disease) were associated with a higher prevalence of SB in older age, and thus might be useful to predict which people will be highly sedentary as an older adult (van der Berg et al. 2014). This will potentially lead to prevention programs, targeted at those people identified, and might reduce SB prevalence in older adults.

Although all the suggestions for both intervention and prevention strategies may have potential, most of them are based on preliminary data only and thus need further investigation to increase evidence and generalizability.

## Conclusion

Based on this review, it can be concluded that older adults are the most sedentary age group, with an accelerometer-derived average daily sitting time of 8.5–9.6 h, representing 65–80 % of their waking time. Although the literature reports negative associations of SB in elderly with outcomes such as less favourable cardio metabolic health, musculoskeletal health, body composition, physical functioning, mental health and QoL, evidence so far is inconclusive apart from the evidence on the adverse effect of SB on the all-cause mortality rate. Prevention of prolonged SB by frequent breaks, while doing at least LIPA, is a promising strategy to counteract adverse health effects. Even though it has not been studied as an intervention in older adults yet, it is expected to be effective on this age group too. This is not only because LIPA appears to be the most common type of PA within the oldest age groups, but also due to the availability of advanced technology. Overall, more studies in elderly are required to increase the evidence level and develop informed SB guidelines including an optimal strategy to counteract SB and its health effects. Nevertheless, the current evidence allows advising and encouraging elderly to limit their SB, as described in the latest physical activity guidelines.
